# Green preparation and characterization of graphene oxide/carbon nanotubes-loaded carboxymethyl cellulose nanocomposites

**DOI:** 10.1038/s41598-018-35984-2

**Published:** 2018-12-04

**Authors:** Yeong-Rae Son, Soo-Jin Park

**Affiliations:** Department of Chemistry, Inha University, 100 Inharo, Incheon, 22212 Republic of Korea

## Abstract

In this study, a homogeneous and stable dispersion of graphene oxide (GO)/carbon nanotube (CNT) complexes (GCCs) was obtained by dispersing CNTs in an aqueous solution using GO in the absence of dispersing agents. Furthermore, carboxymethyl cellulose/GCC (CMC/GCC) nanocomposite films were prepared by a simple solution mixing-evaporation method. The dispersibility of the GCCs with different CNT contents was investigated by UV-Vis spectrophotometry. The morphological and crystalline structures of the samples were analyzed by transmission electron microscopy, scanning electron microscopy, and X-ray diffraction. X-ray photoelectron spectroscopy and Fourier-transform infrared spectroscopy were conducted to identify the chemical composition of GO, CNTs, and GCCs. These results revealed that CNTs could be stably dispersed in water using GO. In addition, when CMC/GCC nanocomposite films were prepared by mixing CMC and GCCs, CNTs were uniformly dispersed in the CMC matrix. The tensile behavior was investigated using a universal testing machine. The tensile strength and Young’s modulus of the CMC/GCC nanocomposite films were significantly improved by up to about 121% and 122%, respectively, compared to those of pure CMC because of uniform and strong π-π interfacial interactions between CNTs and CMC polymer.

## Introduction

Because of the increase in environmental pollution due to non-biodegradable synthetic polymers, such as emissions of toxic gases (e.g., dioxin) by incineration, and the resulting destruction of ecological systems, biodegradable polymers have attracted attention as alternatives for overcoming the limitations of synthetic polymers obtained from petrochemicals. Biodegradable polymers, includ-ing polysaccharides (cellulose, chitin, starch, glycogen, and pectins), polycaprolactone, poly(lactic acid), and polyhydroxy butyrate, have been used in various fields, such as packaging, engineering, and medical applications, because of their biodegradability, biocompatibility, and non-toxicity^1–4^. Carboxymethyl cellulose (CMC) is one of the most important industrial biopolymers. CMC is a semi-synthetic derivative of cellulose produced by partial substitution of the 2, 3, and 6 hydroxyl groups of cellulose by carboxymethyl groups. The structure of CMC is based on the β-(1 → 4)-D-glucopyranose polymer of cellulose. CMC shows polyelectrolyte characteristics because of the pres-ence of weakly acidic carboxymethyl groups^5,6^ and it possesses beneficial properties, such as high water solubility, non-toxicity, and non-allergenicity. Therefore, it has potential applications in coating fluids, binders, textiles, paper, food, drug delivery systems, and cosmetics. Films fabricated from biodegradable polymers like CMC have been widely applied because of their potential proper-ties, such as preservation during storage, superior optical properties, and food protection^7^. Howev-er, these biopolymer-based films show inferior mechanical properties. Therefore, inorganic or hydro-phobic materials, such as clays and carbon materials, have been introduced into biopolymer-based films^8–12^.

Recently, carbon fillers [e.g., graphene oxide (GO), carbon nanotubes (CNTs), and graphene] have been applied to a wide variety of fields, owing to their extraordinary characteristics^13–16^. Graphene (a single atomic layer of graphite with an ideal 2D sp^2^-hybridized structure) and CNTs (cylindrical graphene tubes with a 1D sp^2^-hybridized structure) have been highlighted as reinforcements in polymer matrices because of their many advantages, such as outstanding thermal and mechanical properties, high specific surface areas, chemical stability, and superior electrical conductivity^17–19^. Due to these unique properties, graphene and CNTs are prospective candidates for various applications, such as electronics, sensors, and energy storage and conversion devices. However, graphene-based materials, such as graphene and CNTs, are difficult to utilize as fillers in composites because of some drawbacks, including the restacking problem due to powerful van der Waals forces and poor dispersion stability in water due to hydrophobicity. It is known that graphene-based materials can be dispersed only in a few organic solvents, such as o-dichlorobenzene, N-methylpyrrolidone, N,N-dimethylformamide, and N,N-dimethylacetamide, which are harmful to living organisms and the environment^20–23^. In addition, the dispersion stabilities of graphene and CNTs in these organic solvents are insufficient. There are two main approaches to improve their dispersibility: (1) covalent modifications and (2) non-covalent modifications. Covalent modifications by chemical reactions on the surface of graphene and CNTs, such as oxidation or coupling with diverse functional groups, improve the dispersibility of the materials and can effectively transfer force when mixed in a polymer matrix. However, covalent modifications can change the inherent properties of graphene and CNTs, leading them to lose their original superior properties, such as electrical conductivity and thermal and mechanical characteristics^24–26^. Therefore, non-covalent modifications are applied to maintain the intrinsic properties of graphene and CNTs. They help improve the dispersibility of graphene and CNTs by adsorbing on their surfaces with the use of surfactants (e.g., sodium dodecyl sulfate, sodium cholate, and sodium dodecylbenzenesulfonate) or polymers (e.g., poly(sodium 4-styrene sulfonate) and polyvinyl pyrrolidone). However, mixing of these surfactants and polymers in the polymer matrix may result in the deterioration of mechanical properties^27–30^.

Herein, we successfully disperse CNTs in an aqueous solution using GO. GO, which can be obtained by oxidizing graphite, is an oxidized form of graphene that contains various oxygen-containing functional groups, such as epoxide, carboxyl, and hydroxyl groups^7,31^. There is a non-oxidized and intact sp^2^-hybridized region in the plane of GO. CNTs can be dispersed in aqueous solutions (such as water) by this region without the requirement for any dispersing agents because of the π-π interactions between GO sheets and CNTs^32^. As a result, the GO/CNT complex-reinforced carboxymethyl cellulose (CMC/GCC) nanocomposite films were successfully prepared without using any dispersing agents by mixing a homogeneous dispersion of GO/CNT complexes with CMC. In the present study, GO/CNT complexes in an aqueous solution were investigated, and the characteristics and mechanical properties of the CMC/GCC nanocomposite films were explored.

## Results and Discussion

**Figure 1:**
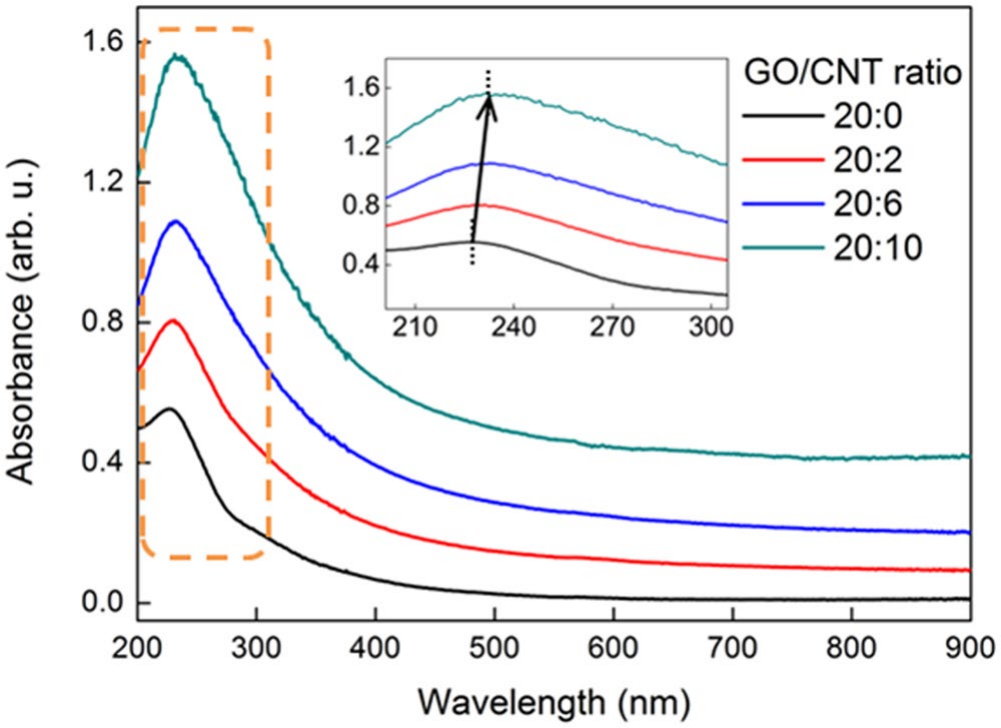
UV-Vis absorbance spectra of GO and GCCs of various weight ratios dispersed in DI water (the inset is a magnification of the dotted box).

The dispersion stability of GO and GCCs with different CNT contents in DI water was analyzed using UV-Vis spectroscopy. To investigate the dispersion stability of GCCs in water, dispersions of 20 mg of GO and various GCCs [20 mg of GO + (2, 6, or 10) mg of CNTs] in 50 mL of DI water were prepared in 70 mL vials using a probe-type ultrasonicator, and the supernatants (2 mL) were diluted with 48 mL of DI water. As shown in Fig. 1, a pronounced main absorption peak and a shoulder peak appeared in the UV-Vis spectrum of GO at 227 and ~300 nm, respectively. These peaks were attributed to the π → π* transitions of aromatic rings (C–C bonds) and the n → π* transitions of carbonyl groups (C=O bonds), respectively. No shift was observed for the shoulder peak of the GCCs at about 300 nm compared to that of GO, indicating that no changes corresponding to the n → π* transitions of the C=O bonds of GO occurred while the GCCs were formed. However, it was confirmed that the main absorption peak of GO at 227 nm underwent a gradual red shift to about 233 nm when the CNT content increased and the absorbance in the whole spectral range increased, indicating electronic conjugation (π–π interactions) between the multiple aromatic regions of the GO sheets and the CNT sidewalls^32^.

**Figure 2:**
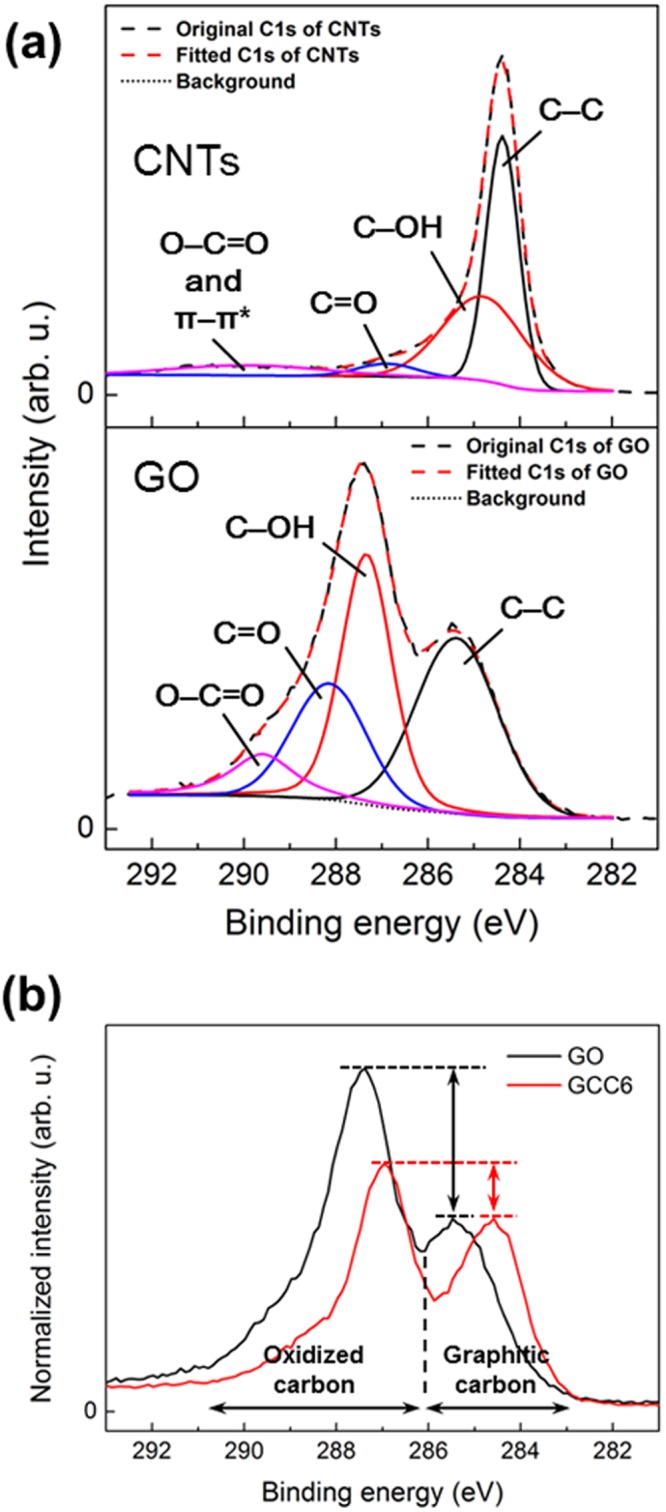
(**a**) Deconvoluted C1s spectra of GO and CNTs and (**b**) normalized C1s spectra of GO and GCC6.

The surface chemistry and crystal structure of GO, CNTs, and GCC6 were investigated by XPS, FT-IR, and XRD. XPS analysis was performed to identify the surface characteristics of GO, CNTs, and GCC6, and their C1s XPS spectra are shown in Fig. 2. As shown in Fig. 2(a), deconvolution of the C1s spectra of GO revealed specific peaks at 285.4 (graphitic carbon, mainly C–C), 287.3 (C–O), 288.2 (C=O), and 289.5 eV (O–C=O), indicating that various oxygen-containing functional groups existed in GO.

The deconvolution of the C1s spectra of CNTs showed different peaks at 284.4 (graphitic carbon, mainly C=C), 284.9 (C–O), 286.7 (C=O), and 290 eV (O–C=O and π–π*), revealing that there were few oxygen functional groups in CNTs. The C1s XPS spectra were normalized with respect to the C–C bonds to compare the relative ratio of oxidized carbon to graphitic carbon. As shown in Fig. 2(b), the C1s XPS spectra of GO and GCC6 exhibited two major binding energies for graphitic carbon (sp^2^- and sp^3^-hybridized carbon) and oxidized carbon at about 284.8 eV and 287 eV, respectively. However, for GCC6, the intensity of the peaks related to oxidized carbon was lower than that for GO due to the intercalated CNTs, which had few or no peaks corresponding to oxidized carbon, as shown in Fig. 2(a).

**Figure 3:**
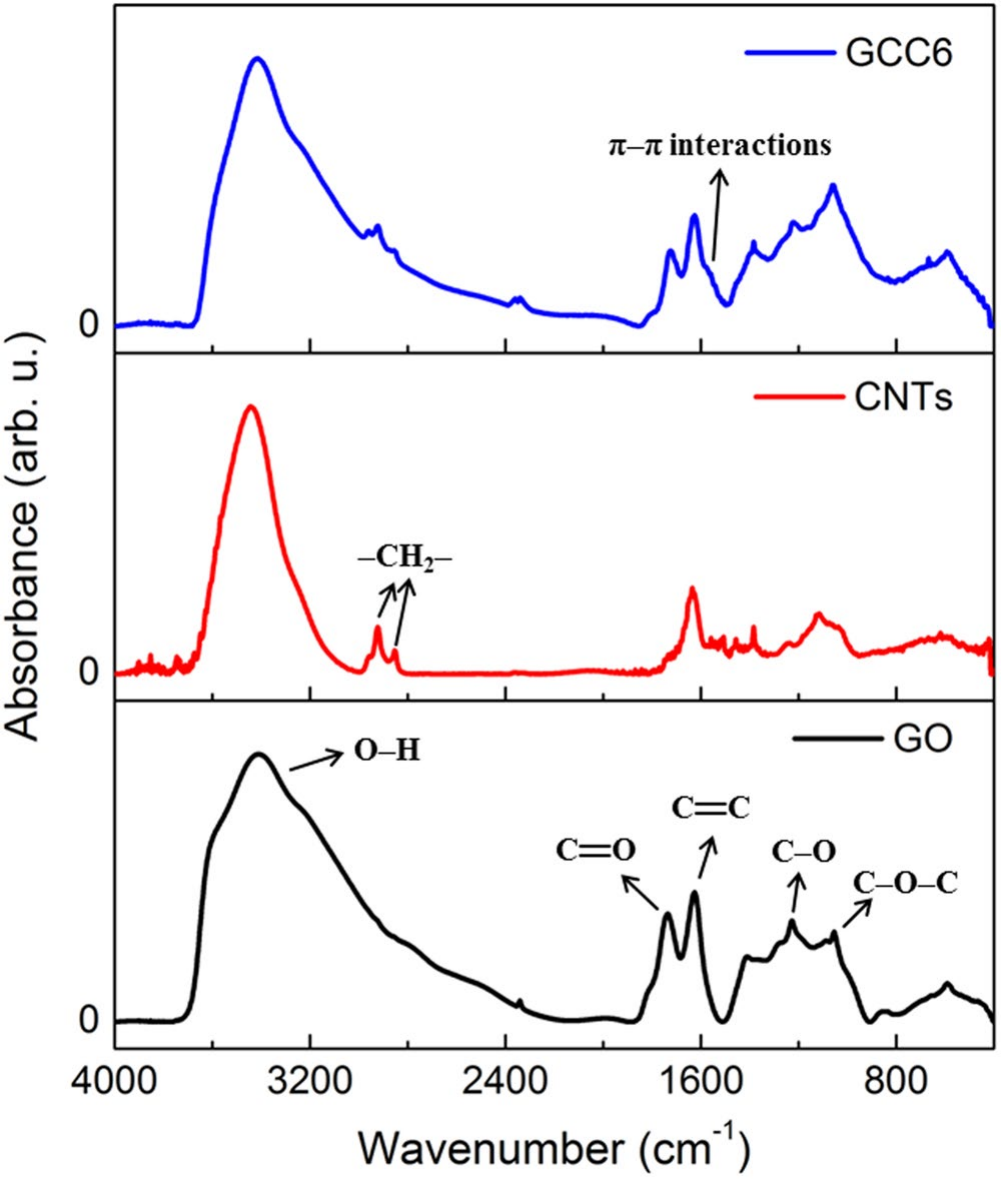
FT-IR spectra of GO, CNTs, and GCC6.

FT-IR spectroscopy was performed to further confirm the qualitative composition of GO, CNTs, and GCC6. As shown in Fig. 3, the following functional groups were confirmed in GO: stretching vibrations corresponding to O–H (3420 cm^−1^), C=O (1730 cm^−1^), C=C (1630 cm^−1^), C–O (1230 cm^–1^), and C–O–C (1057 cm^−1^); and bending vibrations corresponding to O–H in the carboxylic group (1400 cm^−1^). The FT-IR spectrum of the CNTs showed peaks at 2924 cm^−1^ and 2850 cm^−1^ due to the vibration of –CH_2_– bonds in the lattice. In addition, a broad peak was observed at 3420 cm^−1^, which may be attributed to the O–H functional group at the edges of the CNTs. The FT-IR spectrum of GCC6 was similar to that of GO, and two additional peaks corresponding to asymmetric (2925 cm^−1^) and symmetric (2850 cm^−1^) –CH_2_– stretching vibrations originating from CNTs were observed. Furthermore, a shoulder peak was exhibited at 1570 cm^−1^, which could be related to the π–π interactions between GO sheets and the CNTs^33^.

**Figure 4:**
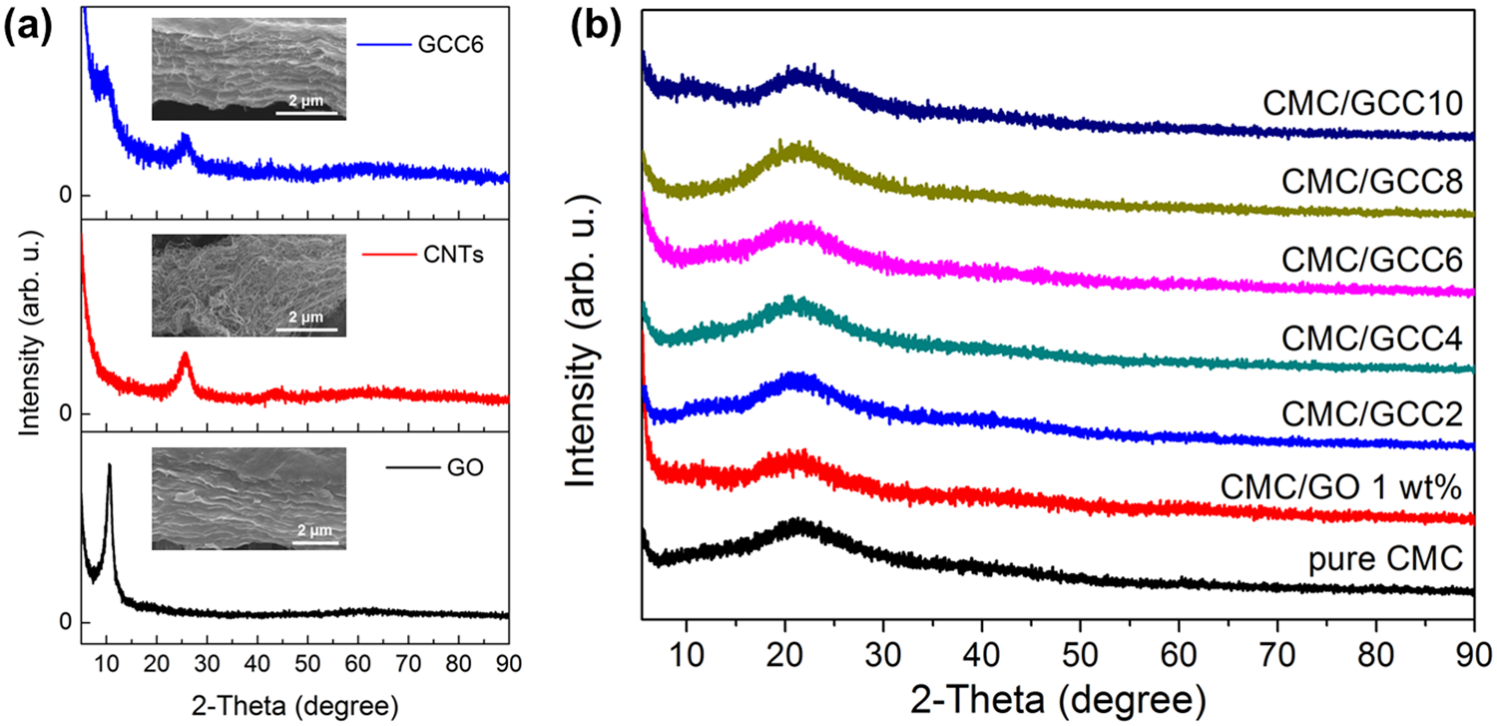
XRD patterns of (**a**) GO, CNTs, and GCC6 and the (**b**) pure CMC, CMC/GO 1 wt%, and CMC/GCC nanocomposite films. The images in (**a**) are the SEM images of the GO, CNTs, and GCC6.

The XRD patterns of the carbon materials (GO, CNTs, and GCC6) and the nanocomposite films (pure CMC, CMC/GO 1.0 wt%, and CMC/GCC with different amounts of CNTs) are shown in Fig. 4(a,b), respectively. The XRD patterns were collected in the 2θ range of 5°–90°. As shown in Fig. 4(a), the typical peaks of GO and CNTs were located at 2θ = 10.56° and 25.83°, respectively, indicating that the interlayer distances of GO and CNTs corresponded to 8.371 Å and 3.446 Å, respectively. Two weak peaks in the XRD pattern of GCC6 appeared at 2θ = 10.56° and 25.83°, corresponding to GO and CNTs, respectively, indicating that the CNTs were located between the GO layers to form complexes. Figure 4(b) shows the XRD patterns of pure CMC, CMC/GO 1 wt%, and CMC/GCC nanocomposite films (GCC2 to GCC10). In the XRD pattern of the pure CMC film, a wide range of peaks was observed at about 2θ = 21°, indicating that its crystal structure was amorphous. After addition of GCC into the CMC matrix, peaks associated with GO and CNTs were not observed, whereas the broad peak of CMC/GCC nanocomposite films at 2θ = 21° was the same as that observed for pure CMC. There was no change in the peak positions. Therefore, the XRD patterns of the CMC/GCC nanocomposite films were comparable to that of the pure CMC film, indicating that the GCCs were dispersed in the CMC matrix. Furthermore, the incorporation of GCC did not affect the amorphous structure of CMC.

**Figure 5:**
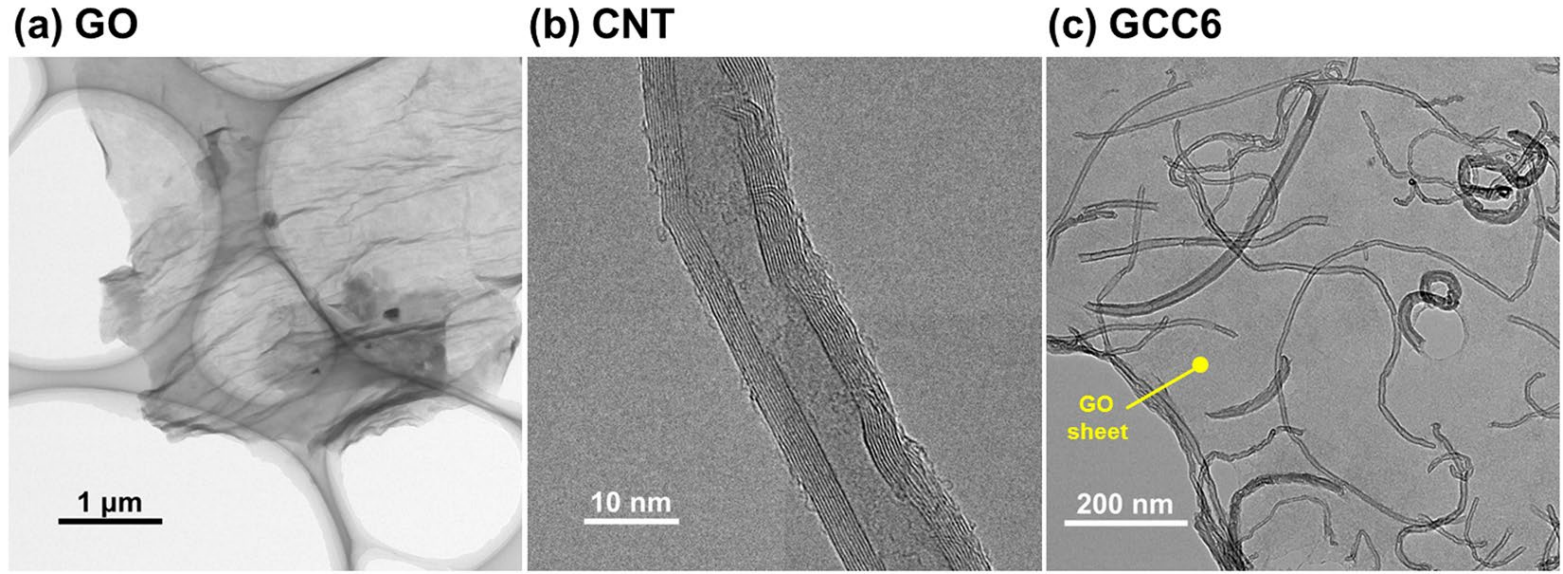
TEM images of GO, CNT, and GCC6 on a holey TEM grid.

TEM observations were performed to observe the external appearance of GO sheets, CNTs, and CNTs dispersed on the GO sheets. As shown in Fig. 5(a–c), the GO sheet, CNTs, and GCC6 on a holey TEM grid were confirmed. The Fig. 5(c) image revealed that they were well linked, with CNTs distributed homogeneously and individually on the matrix of the GO sheets without any aggregation. In addition, the micrograph showed that almost all CNTs were adsorbed on the planes of the GO sheets and not on the edges with hydrophilic groups (mainly carboxylic groups), which was in good agreement with the UV-Vis analysis.

**Figure 6:**
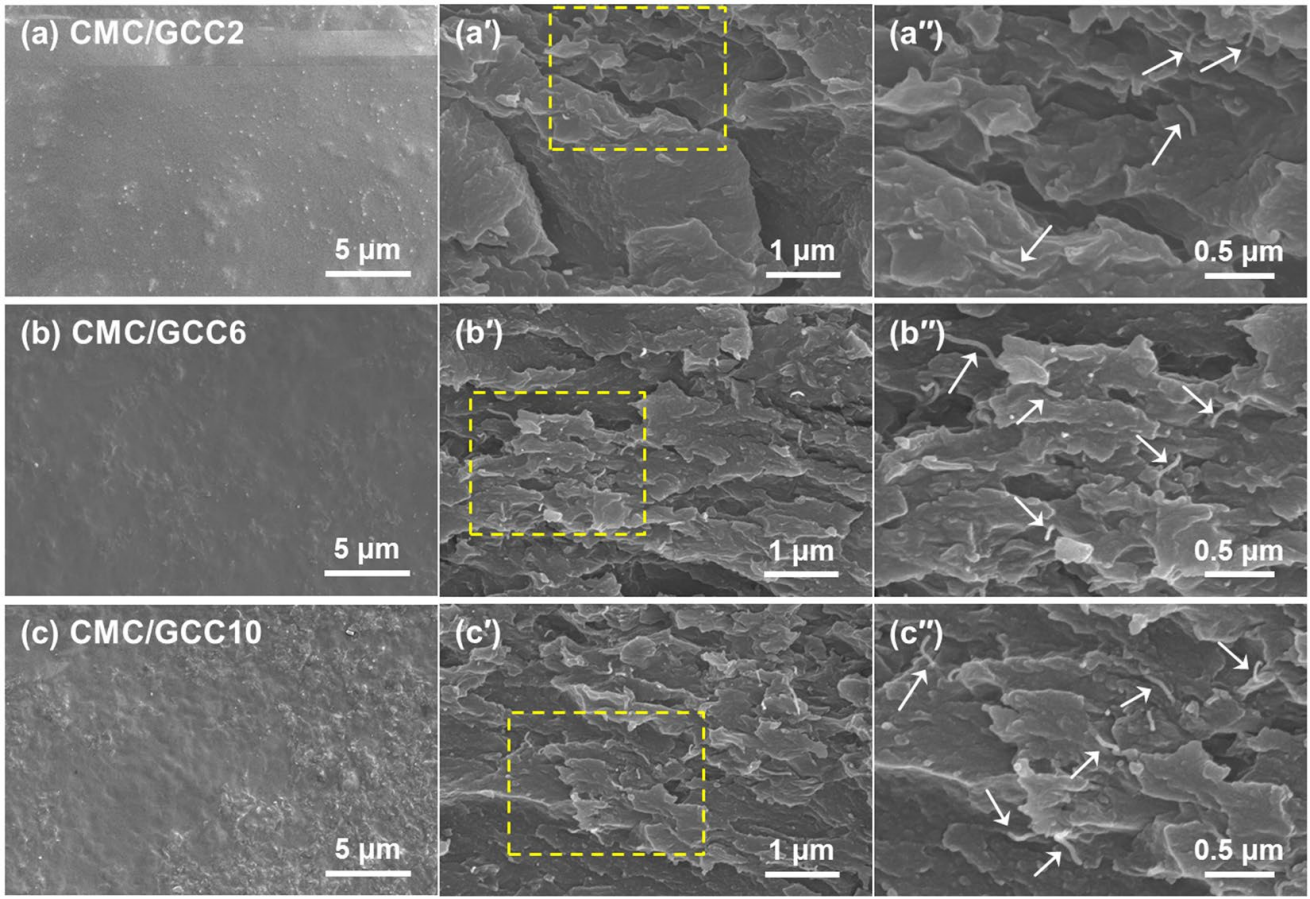
SEM observations of the surfaces and fracture surfaces of nanocomposite films: (**a**) CMC/GCC2, (**b**) CMC/GCC6, and (**c**) CMC/GCC10. (a′), (b′), and (c′) are the fracture surfaces of (**a**), (**b**), and (**c**), respectively. (a′′), (b′′), and (c′′) are the magnified image of yellow dotted box of (a′), (b′), and (c′), respectively. The white arrows in the (b′′), (c′′), and (d′′) indicated the individual CNT.

SEM observations (Fig. 6) were performed to characterize the surfaces and the fracture surfaces of the CMC/GCC nanocomposite films. As shown in Figs S1(a) and 6(a), large CMC agglomerates were confirmed on the surface of the pure CMC film. However, as the CNT content of GCC increased, the CMC agglomerates on the surface of the CMC/GCC nanocomposite films gradually became smaller and almost disappeared [Fig. 6(a–c)].

Furthermore, as shown in Fig. 6(c), it was confirmed that the surface of the film was roughened with ridges. Figs S1(a′) and 6(a′–c′) show the fracture surfaces of the pure CMC and CMC/GCC nanocomposite films, respectively. The fracture surface of the pure CMC film [Fig. S1(a′)] showed an uneven and dimpled structure, representing a typical ductile fracture surface, whereas the fracture surface of the GCC-added CMC nanocomposite films was smooth and less dimpled than that of the CMC film, indicating that the failure mode of the CMC/GCC nanocomposite films changed from ductile to brittle with increasing CNT content in the CMC matrix. The GO sheets in the CMC matrix were difficult to identify clearly, but CNTs were found to be well dispersed without aggregation, leading to efficient load transfer from the CMC matrix to CNTs. Moreover, it was confirmed that the CNTs observed per unit area increased as a function of an increment in CNT content. In addition, the fracture surface of CMC/GCC2 showed a distinct layered structure, which is attributed to the GO sheet. As the content of CNT increased, it was difficult to observe clearly a layered structure. It is considered that the CNTs transfer the load when the film breaks and that cracks are propagated along the CNT surface.

The mechanical behaviors (tensile strength and Young’s modulus) of the CMC/GCC nanocomposite films were confirmed by a UTM. Tensile measurements were performed at room temperature. As shown in Table 1, the mechanical behaviors of the CMC/GO 1 wt% and CMC/GCC nanocomposite films were considerably improved compared to those of the pure CMC film.

**Table 1:** Tensile strength, Young’s modulus, and strain of the pure CMC, CMC/GO 1.0 wt%, and CMC/GCC nanocomposite films (SD indicates standard deviation).

Sample type	Composition (mg)		Tensile strength (MPa)		Young’s modulus (GPa)		Strain (%)	
	GO	CNTs	Mean	SD	Mean	SD	Mean	SD
1	0	0	37.28	1.58	1.89	0.19	11.85	3.51
2	20	0	39.72	2.04	2.14	0.12	11.44	1.80
3	20	2	71.99	3.76	3.56	0.23	6.07	0.63
4	20	4	82.39	3.65	4.11	0.20	4.69	0.78
5	20	6	77.63	3.15	4.20	0.33	4.55	0.55
6	20	8	79.71	0.71	3.79	0.11	4.76	0.53
7	20	10	75.93	2.02	3.94	0.12	6.03	0.59

**Figure 7:**
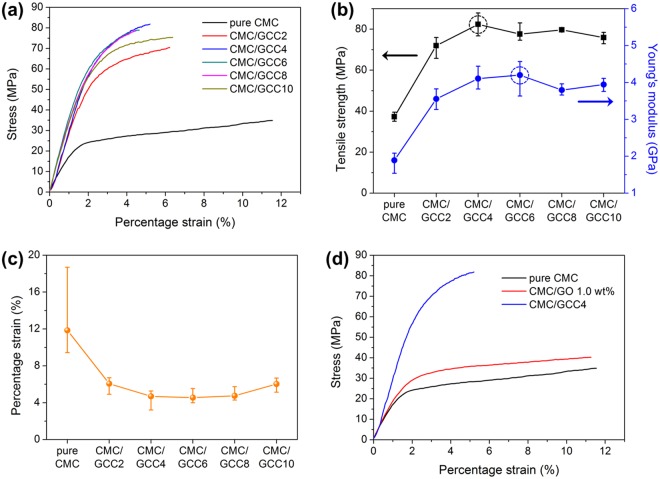
Mechanical behaviors of the pure CMC and CMC/GCC nanocomposite films: (**a**) Stress–strain curves, (**b**) tensile strength and Young’s modulus (dotted circle indicates the maximum point of each mechanical property), (**c**) strain to failure (each data point is the average of replicate measurements and error bars indicate minimum and maximum values), and (**d**) stress–strain curves of the pure CMC, CMC/GO 1.0 wt%, and CMC/GCC4 nanocomposite films.

As shown in Fig. 7(a), the stress initially increased almost linearly with the strain. Nonlinear behavior was revealed for all films from yield stress to maximum stress. As shown in Table 1 and Fig. 7(b), the tensile strength and Young’s modulus of the CMC/GCC2 nanocomposite film increased significantly compared with those of the pure CMC and CMC/GO 1 wt% films. However, the strain decreased because of the transition of the failure mode from ductile to brittle with the addition of CNTs. In pure CMC with a ductile nature, a load is randomly transferred by the randomly arranged CMC polymer chain. When CNTs are added to a CMC matrix with such ductile characteristics, a load is directed along the surface of the CNT. Therefore, when a crack is generated, the crack propagates rapidly in the direction of the load transfer, resulting in a low ductility. The tensile strengths of the CMC nanocomposite films with various CNT contents (from GCC2 to GCC10) were 71.99, 82.39, 77.63, 79.71, and 75.93 MPa, respectively. Young’s modulus was measured at 3.56, 4.11, 4.20, 3.79, and 3.94 GPa, respectively. The maximum tensile strength and Young’s modulus were 121% and 122% higher than those of the pure CMC film and 107% and 96% higher than those of the CMC/GO 1 wt% film, respectively. However, as shown in Fig. 7(b,c), the CMC/GCC10 nanocomposite film showed a Young’s modulus lower than that of CMC/GCC6 and increased strain. The morphology of the GCC became crumpled with increasing the CNT content. As shown in Fig. 6(c), the GCC10 with a crumpled structure allowed the surface of the CMC/GCC10 nanocomposite film to have a corrugated shape. As illustrated in Fig. S3(c), when a load is applied to the film, the load is transferred to the GCC and deformation occurs in the GCC having the crumpled structure. It is believed that the deformation of the GCC increases the ductility of the film compared to that of CMC/GCC nanocomposite films with low/moderate CNT content. Furthermore, because the GCC has a crumpled and aggregated morphology due to a high amount of CNT, the load is not as effectively transferred to the GCC. Therefore, it is considered that the tensile strength of the CMC/GCC10 nanocomposite film is relatively lower than that of the CMC/GCC nanocomposite film with a low/moderate amount of CNT.

The typical stress-strain curves of the pure CMC, CMC/GO 1 wt%, and CMC/GCC4 nanocomposite films are shown in Fig. 7(d), indicating that even at low CNT contents, the mechanical behaviors of the nanocomposites were significantly enhanced. The improvement in the tensile strength of the CMC/GO 1 wt% film was due to the hydrogen bonding between CMC and the oxygen functional groups in the GO sheets. In addition, it was known that the enhancement in mechanical properties by the addition of CNTs to the polymer matrix is generally due to strong interfacial interactions, such as π–π stacking, between the polymer chains and the CNT surface. In order to assess the π–π interfacial interaction between CNT and CMC, UV-Vis spectroscopy was performed. As shown in Fig. S2, two broad peaks appeared in the UV-Vis spectra of CNT in IPA at around 233.4 nm (main) and 260.3 nm (shoulder). The broad peaks have been attributed to the π plasmon resonances, collective excitations of π electrons, in the free electrons of CNTs^34^. The broad absorption peaks were also observed in the CNTs in water dispersed by CMC, and notably, the shoulder absorption peak was red-shifted from 260.3 to 263.9 nm. Furthermore, the absorption intensity of the shoulder peak was increased compared to that of CNT in IPA. This result suggests that a π–π interfacial interaction was formed between CNTs and CMC, resulting in an increase of anchoring of CMC molecules at the CNT wall^35^. In this study, therefore, the mechanical properties of CMC were improved by the presence of only a very small amount of CNTs compared to GO and CMC contents.

In summary, CMC/GCC nanocomposite films were prepared by mixing a CMC solution and GCC dispersions in DI water. A homogeneous dispersion of GCCs in an aqueous solution was obtained by a simple procedure: mixing and sonication. The dispersibility of the GCCs was confirmed by UV-Vis spectroscopy, indicating that the CNTs could be dispersed due to the π–π interactions between the sidewalls of the CNTs and GO sheets. In addition, TEM investigations confirmed that the CNTs were individually distributed on the surface of the GO sheets. After the GCC dispersion and the CMC solution were mixed and dried to produce CMC/GCC nanocomposite films, XRD and SEM revealed that the GCCs were uniformly dispersed in the CMC matrix. The overall mechanical properties of the CMC/GCC nanocomposite films were significantly improved compared to those of the pure CMC and CMC/GO 1 wt% films. Consequently, the CNTs could be dispersed in water without using harmful solvents and modification approaches that deteriorate the inherent properties of CNTs. Thus, the mechanical properties of CMC, a biodegradable polymer, can be significantly improved by adding only a small amount of CNTs. It is expected that these films can be applied in a green and effective manner to not only composite materials but also various fields, such as electronics, sensors, and energy storage and conversion.

## Materials and Methods

### Materials

Sodium carboxymethyl cellulose (CMC, average M  ~ 250,000) and graphite flakes were purchased from Sigma Aldrich Korea. Sulfuric acid (H_2_SO_4_; 98%) and phosphoric acid (H_3_PO_4_; 85%) were purchased from DAEJUNG and DUKSAN, respectively. Potassium permanganate (KMnO_4_; 99.3%) and hydrogen peroxide (H_2_O_2_; 30%) were purchased from OCI Company Ltd., Korea. Hydrochloric acid (HCl; 35%–37%) and anhydrous ethyl alcohol (99.5%) were purchased from SAMCHUN and DAEJUNG, respectively. CNTs were supplied by Nanosolution Co., Korea.

### Synthesis of graphene oxide (GO)

GO was synthesized from graphite flakes using oxidants and acids^36,37^. First, 3 g of graphite flakes were added into a mixed solution of 360 mL of concentrated H_2_SO_4_ and 40 mL of concentrated H_3_PO_4_ under stirring in an ice bath. Next, 18 g of KMnO_4_ was slowly added to the graphite-acid mixture above, and the mixture was reacted overnight at 50 °C in an oil bath. Subsequently, the mixture was cooled to room temperature naturally and poured slowly onto an ice block of approximately 400 mL while being stirred. Then, 3 mL of hydrogen peroxide was added gradually to the mixture to terminate the reaction, and the color of the mixture became light brown. To neutralize the reacted suspension, it was washed twice with deionized (DI) water, 10% HCl solution, and anhydrous ethanol via centrifugation (8000 rpm for 30 min), and it was washed successively with DI water until the pH reached above 6. Finally, after the suspension was sonicated mildly using an ultrasonic bath for 30 min, GO powder was obtained by freeze drying and was dried under vacuum at room temperature for 6 h.

### Preparation of GO/CNT complex-reinforced CMC nanocomposite films

**Figure 8:**
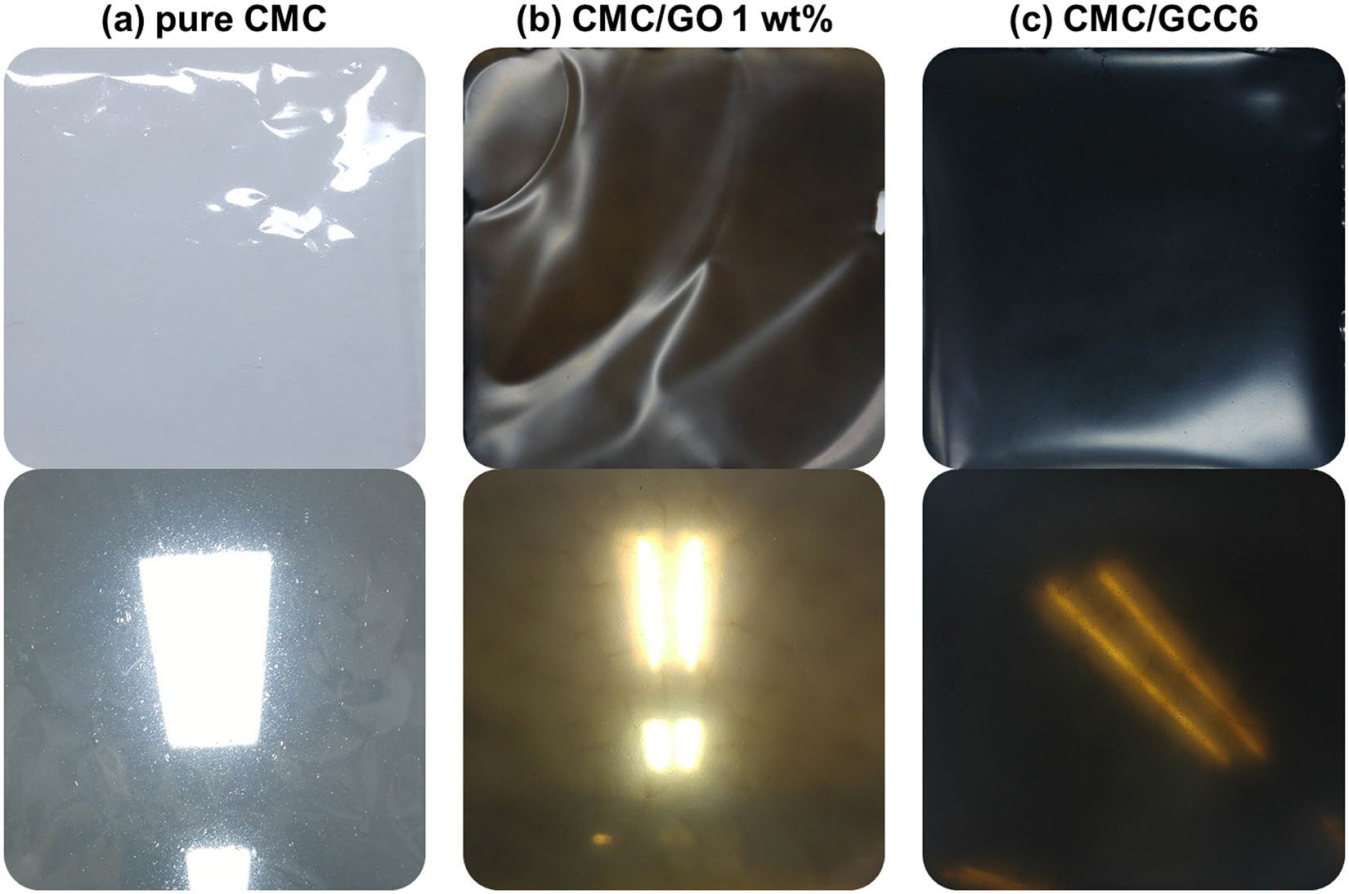
Digital photographs of (**a**) pure CMC, (**b**) CMC/GO 1 wt%, and (**c**) CMC/GCC6 nanocomposite films.

Before describing the preparation process of the nanocomposite films, it should be noted that GCCn (n = 2, 4, 6, 8, 10) refers to GO/CNT complexes produced by mixing 20 mg of GO and n mg of CNTs. GO/CNT-reinforced CMC (CMC/GCC) nanocomposite films were prepared by a simple solution mixing-evaporation method^7^. The CMC/GCC6 nanocomposite film was prepared as follows: a solution of 1.98 g of CMC in 49 mL of DI water was stirred for 12 h to completely dissolve the CMC. Then, 20 mg of GO and 6 mg of CNTs were placed in a vial containing 50 mL of DI water and stirred for about 3 h. The mixture was then sonicated by a probe-type ultrasonicator for 1.5 h in an ice bath, resulting in a GCC6 dispersion. The GCC6 dispersion was then mixed with the CMC solution, followed by stirring for 12 h. Subsequently, a silicon rubber sheet frame was fixed onto a glass plate, and the prepared solution was poured into the frame. The glass plate containing the solution was placed in a vacuum oven and dried at 80 °C under vacuum until the film was formed. The dried CMC/GCC6 nanocomposite thin film (average thickness: 0.08 mm) was carefully detached from the glass plate. CMC/GCC nanocomposite films with different CNT contents were also prepared by identical procedures. Pure CMC, CMC/GO 1 wt% prepared by the same procedure using 20 mg of GO, and the CMC/GCC6 nanocomposite films are shown in Fig. 8(a–c), respectively.

### Characterization of samples

UV-Vis spectra were measured using a UV-Vis spectrophotometer (Scinco S-3100) to confirm the dispersibility of GO/CNT complexes (GCCs) in water. Transmission electron microscopy (TEM, Philips CM200) observations were performed to investigate the GO sheets, CNTs, and GCCs. The morphological structure of the surface and the fracture surface of the as-prepared pure CMC film and nanocomposite films of CMC/GO 1 wt% and CMC/GCC were investigated using scanning electron microscopy (SEM, Hitachi S-4300). X-ray diffraction (XRD) analysis of the crystal structure of the prepared samples was performed with a D2 PHASER (Bruker) instrument with a Lynx-Eye detector using Cu Kα radiation at 30 kV and 10 mA (λ = 1.5406 Å). X-ray photoelectron spectroscopy (XPS, Thermo Scientific K-Alpha) was used to quantitatively analyze the chemical compositions of GO, CNTs, GCC6, the pure CMC film, and the nanocomposite films of CMC/GO 1 wt% and CMC/GCC. Fourier transform infrared vacuum spectrometry (FT-IR, Vertex 80 V) was used to identify the functional groups of GO, CNT, and GCCs and was recorded in the range of 400–4000 cm^−1^. The mechanical properties of the CMC nanocomposite films were measured according to ASTM D638-05 using a universal testing machine (UTM, LR5KPlus, LLOYD Instruments Ltd.) at a loading rate of 2 mm min^−1^ with a gauge length of 25 mm. The measured tensile strength, Young’s modulus, and the strain were averaged for 5 specimens.

## Electronic supplementary material

Supporting Information

## References

[1] Park SB, Lih E, Park KS, Joung YK, Han DK (2017). Biopolymer-based functional composites for medical applications. Prog. Polym. Sci..

[2] Wang Y, Ameer GA, Sheppard BJ, Langer R (2002). A tough biodegradable elastomer. Nat. Biotechnol..

[3] Jin FL, Pang QQ, Zhang TY, Park SJ (2015). Synergistic reinforcing of poly(lactic acid)-based systems by polybutylene succinate and nano-calcium carbonate. J. Ind. Eng. Chem..

[4] Park SJ, Jin FL, Lee JR (2004). Effect of biodegradable epoxidized castor oil on physicochemical and mechanical properties of epoxy resins. Macromol. Chem. Phys..

[5] Chakraborty T, Chakraborty I, Ghosh S (2006). Sodium Carboxymethylcellulose-CTAB Interaction: A Detailed Thermodynamic Study of Polymer-Surfactant Interaction with Opposite Charges. Langmuir.

[6] Yadav M, Rhee KY, Jung IH, Park SJ (2013). Eco-friendly synthesis, characterization and properties of a sodium carboxymethyl cellulose/graphene oxide nanocomposite film. Cellulose.

[7] Son YR, Rhee KY, Park SJ (2015). Influence of reduced graphene oxide on mechanical behaviors of sodium carboxymethyl cellulose. Compos. Pt. B-Eng..

[8] Han DL, Yan L, Chen W, Li W (2011). Preparation of chitosan/graphene oxide composite film with enhanced mechanical strength in the wet state. Carbohydr. Polym..

[9] Mittal G, Dhand V, Rhee KY, Park SJ, Lee WR (2015). A review on carbon nanotubes and graphene as fillers in reinforced polymer nanocomposites. J. Ind. Eng. Chem..

[10] Ha SR, Ryu SH, Park SJ, Rhee KY (2007). Effect of clay surface modification and concentration on the tensile performance of clay/epoxy nanocomposites. Mater. Sci. Eng. A-Struct..

[11] Yim YJ, Park SJ (2015). Electromagnetic interference shielding effectiveness of high-density polyethylene composites reinforced with multi-walled carbon nanotubes. J. Ind. Eng. Chem..

[12] Hwang SY, Yoo ES, Im SS (2012). The synthesis of copolymers, blends and composites based on poly(butylene succinate). Polym. J..

[13] Wang Y, Voronin GA, Zerda TW, Winiarski A (2006). SiC–CNT nanocomposites: high pressure reaction synthesis and characterization. J. Phys.: Condens. Matter.

[14] Gubicza J (2006). Microstructure of diamond–SiC nanocomposites determined by X-ray line profile analysis. Diam. Relat. Mat..

[15] Wang Y, Zerda TW (2006). The mechanism of the solid-state reaction between carbon nanotubes and nanocrystalline silicon under high pressure and at high temperature. J. Phys.: Condens. Matter.

[16] Wang Y, Zerda TW (2007). Microstructure evaluations of carbon nanotube/diamond/silicon carbide nanostructured composites by size–strain line-broadening analysis methods. J. Phys.: Condens. Matter.

[17] Kim KS, Park SJ (2011). Influence of multi-walled carbon nanotubes on the electrochemical performance of graphene nanocomposites for supercapacitor electrodes. Electrochim. Acta.

[18] Zhu Y (2010). Graphene and graphene oxide: synthesis, properties, and applications. Adv. Mater..

[19] Lee SY, Chong MH, Rhee KY, Park SJ (2014). Silver-coated graphene electrode produced by electrolytic deposition for electrochemical behaviors. Curr. Appl. Phys..

[20] Fujigaya T, Nakashima N (2015). Non-covalent polymer wrapping of carbon nanotubes and the role of wrapped polymers as functional dispersants. Sci. Technol. Adv. Mater..

[21] Ausman KD, Piner R, Lourie O, Ruoff RS (2000). Organic solvent dispersions of single-walled carbon nanotubes: toward solutions of pristine nanotubes. J. Phys. Chem. B..

[22] Furtado CA (2004). Debundling and dissolution of single-walled carbon nanotubes in amide solvents. J. Am. Chem. Soc..

[23] Park S (2009). Colloidal suspensions of highly reduced graphene oxide in a wide variety of organic solvents. Nano Lett..

[24] Rai PK (2006). Isotropic-nematic phase transition of single-walled carbon nanotubes in strong acids. J. Am. Chem. Soc..

[25] Kovtyukhova NI, Mallouk TE, Pan L, Dickey EC (2003). Individual single-walled nanotubes and hydrogels made by oxidative exfoliation of carbon nanotube ropes. J. Am. Chem. Soc..

[26] Coleman JN, Khan U, Gun’ko YK (2006). Mechanical reinforcement of polymers using carbon nanotubes. Adv. Mater..

[27] Lotya M, King P, Khan U, De S, Coleman J (2010). High-concentration, surfactant-stabilized graphene dispersions. ACS Nano.

[28] Pu NW (2012). Dispersion of graphene in aqueous solutions with different types of surfactants and the production of graphene films by spray or drop coating. J. Taiwan Inst. Chem. E..

[29] Shin MK (2012). Synergistic toughening of composite fibres by self-alignment of reduced graphene oxide and carbon nanotubes. Nat. Commun..

[30] Tkalya E, Ghislandi M, With G, Koning C (2012). The use of surfactants for dispersing carbon nanotubes and graphene to make conductive nanocomposites. Curr. Opin. Colloid. In..

[31] Kim HJ (2017). Maximizing volumetric energy density of all-graphene-oxide supercapacitors and their potential applications for energy harvest. J. Power Sources.

[32] Zhang C, Ren L, Wang X, Liu T (2010). Graphene oxide-assisted dispersion of pristine multiwalled carbon nanotubes in aqueous media. J. Phys. Chem. C.

[33] Wang J (2014). One-Pot Synthesis of CdS–Reduced Graphene Oxide 3D Composites with Enhanced Photocatalytic Properties. Cryst. Eng. Comm..

[34] Rance GA, Marsh DH, Nicholas RJ, Khlobystov AN (2010). UV–vis absorption spectroscopy of carbon nanotubes: Relationship between the π-electron plasmon and nanotube diameter. Chem. Phys. Lett..

[35] Kim A, Park SJ, Lee JR (1998). Stabilization of Liquid Crystal-in-Water Dispersion with Polymer/Surfactant Mixture: Nematic Curvilinear Aligned Phase Composite Film. J. Colloid Interface Sci..

[36] Hummers WS, Offeman RE (1958). Preparation of graphitic oxide. J. Am. Chem. Soc..

[37] Marcano DC (2010). Improved synthesis of graphene oxide. ACS Nano.

