# Supplementary material for: Green preparation and characterization of graphene oxide/carbon nanotubes-loaded carboxymethyl cellulose nanocomposites
Source: Sci Rep. 2018 Dec 4;8:17601. doi: 10.1038/s41598-018-35984-2 (PMC6279817; doi:10.1038/s41598-018-35984-2)
Supplement: Supplementary file 1 — Supporting Information [file 41598_2018_35984_MOESM1_ESM.docx]

**Supplementary Information**

**Green preparation and characterization of graphene oxide/carbon nanotubes-loaded carboxymethyl cellulose nanocomposites**

**Yeong-Rae Son and Soo-Jin Park***

*Department of Chemistry, Inha University, 100 Inharo, Incheon 22212, Republic of Korea*

*Corresponding author. Tel.: +82-32-860-8438; Fax: +82-32-860-8438.

E-mail address: sjpark@inha.ac.kr (S.-J. Park)

**Supporting Figures**


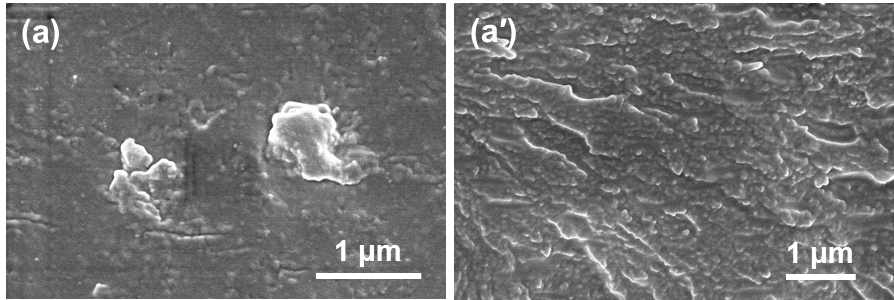


**Fig. S1.** SEM observations of (a) the surfaces and (a′) the fracture surfaces of pure CMC film.


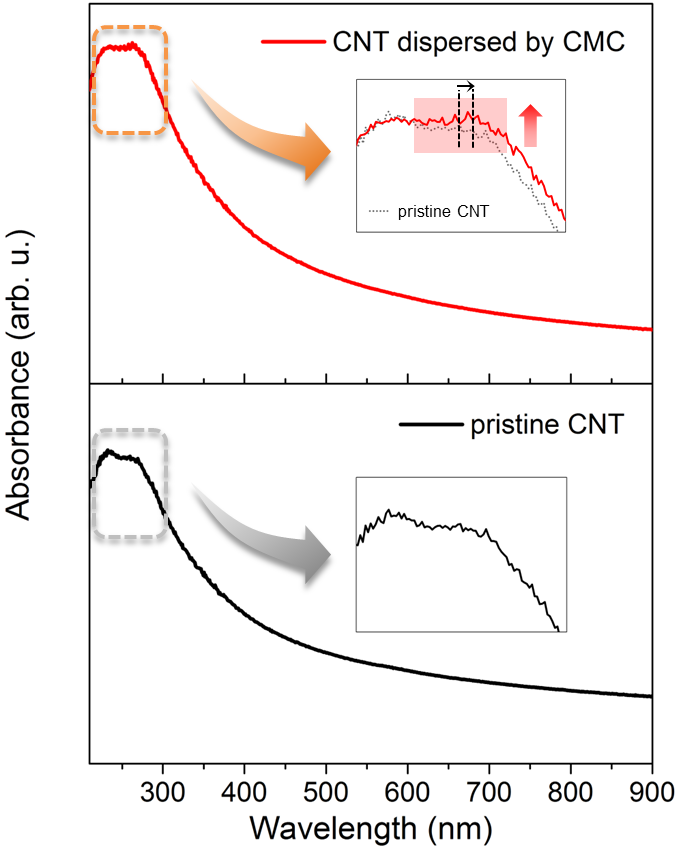


**Fig. S2.** UV-Vis spectra of CNT dispersed in water by CMC and in isopropyl alcohol (IPA).

0.05 mg/mL of CNT dispersion in water and IPA was used for UV-Vis analysis. Each measurement was investigated after measuring the base line with corresponding solvent. The ratio of CNT to CMC dispersed in water was 1:1. The UV-Vis spectra were recorded in the range of 220–900 nm.


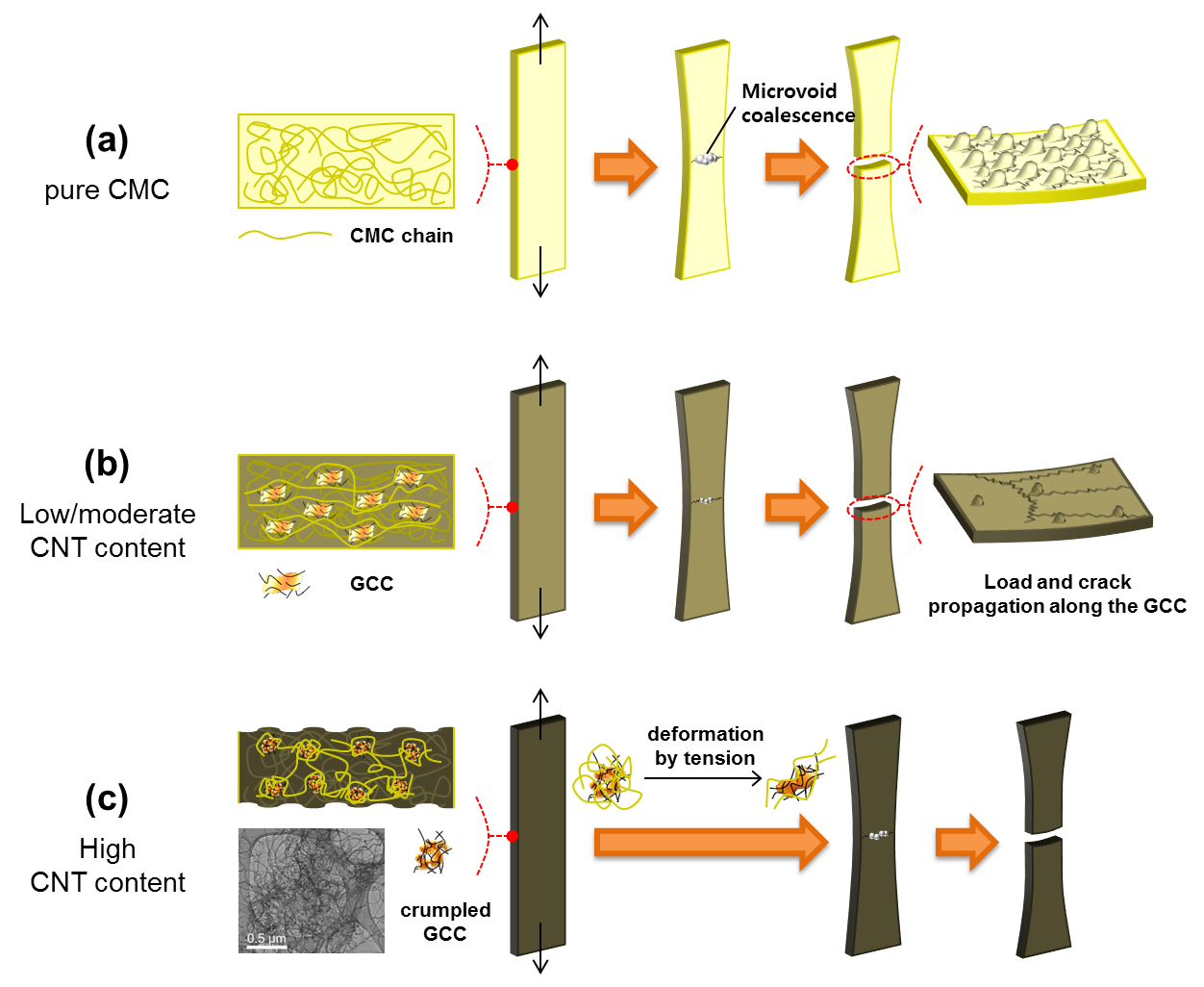


**Fig. S3.** Schematic explanation of deformation mechanism of CMC/GCC nanocomposite films with CNT addition.
